# Dietary Supplements with Proline—A Comprehensive Assessment of Their Quality

**DOI:** 10.3390/life13020263

**Published:** 2023-01-18

**Authors:** Krzysztof Adam Stępień, Weronika Krawczyk, Joanna Giebułtowicz

**Affiliations:** Department of Drugs Chemistry, Faculty of Pharmacy, Medical University of Warsaw, 1 Banacha, 02-097 Warsaw, Poland

**Keywords:** dietary supplement, proline, food supplement analysis, dissolution test, quality control

## Abstract

Dietary supplements are food products commonly used worldwide to obtain nutritional and physiological effects. They can contain a wide variety of active substances and can be administered for health and disease. Their use can be beneficial if justified, and their quality is adequate. Unfortunately, data on the quality of supplements is scarce. As part of this work, we assess the quality of seven dietary supplements containing proline. The preparations were produced in the EU and the USA. The quality assessment consisted of the detection of potential impurities, the determination of the content of the main ingredient, and the release of proline. The technique used to analyse impurities and proline (Pro) content was liquid chromatography coupled with tandem mass spectrometry. We detected five contaminants. The main ingredient content was in the range of 73–121% in capsules and 103–156% in tablets. Five of the seven analysed dietary supplements released below 80% Pro (for each tablet/capsule at pH 1.2). One of the supplements may be inactive because a very low release of Pro was reported. The results, we hope, will increase consumer awareness of the quality of these preparations and result in a change in the regulations governing the marketing of these preparations, at least by making release testing mandatory.

## 1. Introduction

Dietary supplements are growing in popularity worldwide due to the increasing need for health and body care [1]. Using these products is equated with a safe and easy way to live a healthy lifestyle [2]. Dietary supplements are intended to correct nutritional deficiencies and maintain an adequate intake of certain nutrients to produce a nutritional or physiological effect. They may contain vitamins and minerals, plant ingredients and extracts, omega-3 fatty acids [3], probiotics and prebiotics [4], and amino acids [5]. Consumers use dietary supplements prophylactically or in the course of a specific disease. Depending on the intended use, dietary supplements can be divided into those that support weight loss [6], the immune system [7], nervous system [8], cardiovascular system [9], digestive system [10], skin, hair, nails [11], and reducing the risk of osteoporosis [12].

Dietary supplements are often sold in tablet and capsule form, resembling medicinal products [13]. However, in the EU, they are classified as food [14], so their quality is not subject to the exact requirements of drugs [15]. Recently, some reports appeared on the inadequate quality of dietary supplements. The low quality of these preparations may be a consequence of impurities [16]. Contaminants such as heavy metals [17], mycotoxins [18], insecticides [19], herbicides [20], degradation products of the main ingredient [21], and active substances such as sibutramine [22] or sildenafil [23] have often been detected in such products. Another aspect of low-quality supplements is the incorrect content of active ingredients, as shown for tryptophan [21] or melatonin [24]. In addition, if supplements are in the form of tablets or capsules, consumers expect that the substance contained in them can be released from this form at an appropriate level, thus guaranteeing the possibility of obtaining the right effect [25]. Unfortunately, since the manufacturer is not required to conduct a release study, the release of the active substance is sometimes shallow. Then there is no possibility that the substance contained in the supplement will have any effect on the consumer. Low release of the substance contained in the supplement has been described in the case of supplements with tryptophan [21], lutein [26], melatonin [27], and folic acid [28]. However, the amount of data on the quality of supplements is still scarce. For instance, the release of the active substance was analysed for only a few types of supplements. In many cases, a single supplement was analysed [29,30]. The analysis of more than five supplements of one kind was carried out only for calcium carbonate [31], melatonin [27], folic acid [32], lutein [26] and tryptophan [21]. There are even fewer studies in which a comprehensive assessment was made: content, release, and impurities; we found such data only for tryptophan [21].

Since the quality of dietary supplements seems to be a crucial aspect in the context of consumers’ widespread use, dietary supplements containing proline were analysed in this study. Pro is one of the 20 essential L-α-amino acids that build proteins [33], playing a crucial role in building the collagen chain. It participates in the construction of collagen [34]. Collagen’s polypeptide chains are made of repeating Gly-X-Y triples, where any amino acid can occupy the X and Y positions. However, Pro and its derivative, 4-hydroxyproline, are by far the most common, accounting for about 20% of collagen [35]. Pro is also present in the central nervous system [36], and participates in redox balance, affecting the survival or death of cells and cancer development, constituting an element of their so-called metabolic re-programming [37]. Diseases caused by congenital deficiency of enzymes involved in Pro metabolism are relatively rare [38]. However, they clearly illustrate the multidirectional function of Pro and the complexity of its metabolic pathways, which still hide interdependencies that are not fully explained.

The role of Pro in collagen synthesis is why manufacturers of dietary supplements recommend it for use by physically active people who take care of their hair, skin, and nails.

So far, data on the quality of dietary supplements containing Pro are lacking. This study aimed to fill this gap by assessing the quality of dietary supplements containing Pro in tablet or capsule form and manufactured in the EU and the US. All supplements with Pro that can be purchased in Poland were included in the study (*n* = 7). This assessment was covered by performing qualitative analysis of Pro and unknown screening to detect contaminants. Additionally, we assessed the release of Pro from tablets or capsules. In all tests, the most reliable analytical method was applied: liquid chromatography coupled with mass spectrometry. We believe that the results of our study will be an essential argument in any discussion of dietary supplements.

## 2. Materials and Methods

### 2.1. Samples

We included in our study seven dietary supplements with Pro in capsules (Pro1-Pro6) or tablets (Pro7). Supplements were manufactured in Poland (L-Prolina; Biocaps COLLAGEN, Prolina), Germany (L-Prolina), and the US (Włosy, Skóra, Paznokcie; L-Proline). Supplements were purchased in Polish pharmacies, online pharmacies, or on an online e-commerce platform.

### 2.2. Reagents

L-Pro (≥98%) (standard) was purchased from LGC (Luckenwalde, Germany). Sodium hydroxide (≥98.8%), hydrochloric acid (35–38%), and potassium phosphate monobasic (≥99.5%) we bought from Chempur (Piekary Śląskie, Poland). HPLC-grade acetonitrile, formic acid, and methanol were from Merck (Darmstadt, Germany).

### 2.3. Sample Preparation

The sample was prepared based on Stepien and Giebultowicz [21]. Briefly, for each supplement, we randomly selected three tablets or capsules, determined the total weight of the tablets’ or capsules’ content. The tablets were ground into a fine powder in a mortar. Next, we weighed the capsule content or tablet’s mass equivalent to 10 mg of Pro, and added 1.00 mL mixture containing acetonitrile, methanol, and water in a ratio of 1:1:1; *v*/*v*/*v*. Then, the mixture was sonicated (15 min) and centrifuged (5 min). The supernatant was diluted with the mobile phase to a concentration of 500 ng/mL or 100 ng/mL for qualitative and quantitative analysis.

### 2.4. Qualitative Analysis

Instrumental analysis was performed using a UHPLC Dionex Ultimate 3000 with a Q-Exactive spectrometer as described previously [21]. Briefly, the HESI source was operated in positive mode over *m*/*z* 100–1400 with a resolution of 70,000 (*m*/*z* 200). The collision energies were set to 20, 35, and 50 eV.

An Accucore C-18 column (100 mm × 4.6 mm, 2.6 µm, Thermo Fisher Scientific, Waltham, MA, USA) equipped with a security guard was applied for chromatographic separation. The mobile phases consisted of eluent A (0.1% formic acid) and eluent B (acetonitrile with 0.1% formic acid). The gradient (% B) was as follows: 0 min 10%; 1 min 10%; 10 min 95%; 15 min 95%, 16 min 10%; 17 min 10%. The injection volume was 10 µL, the column temperature was 40 °C, and the mobile phase flow rate was 0.3 mL/min.

The data were analyzed using Compound Discoverer 3.3 software (Thermo Fisher Scientific, Waltham, MA, USA).

### 2.5. Quantitative Analysis

Agilent 1260 Infinity (Agilent Technologies, Santa Clara, CA, USA), coupled to a QTRAP 4000 hybrid triple quadrupole/linear ion trap mass spectrometer (AB Sciex, Framingham, MA, USA), was used for quantitative analysis. The turbo ion-spray source was operated in positive mode. The curtain gas, ion source gas 1, ion source gas 2, and collision gas (all high-purity nitrogen) were set at 0.24 MPa, 0.41 MPa, 0.28 MPa, and “medium” instrument units, respectively. The ion spray voltage and source temperature were 5500 V and 600 °C, respectively. The target compounds were analysed in multiple reaction monitoring modes using the 116.0/67.9 transition. We optimized the declustering potential (DP = 56 V), collision energy (CE = 41 V), entrance potential (EP = 10 V), and collision exit potential (CXP = 10 V). The samples were injected at a volume of 10 μL.

A Kinetex C18 column (100 mm × 3.0 mm, 2.6 μm, Phenomenex, Milford, USA) was used for separation. The mobile phases consisted of eluent A (0.1% formic acid) and eluent B (methanol with 0.1% formic acid). The gradient (%B) was: 0 min 10%; 1 min 10%; 2 min 70%; 3 min 70%; 4 min 10%; 5 min 10%. The mobile phase flow rate was 0.5 mL/min.

The concentrations of Pro in all analysed samples were within the range of the calibration curve, i.e., from 0.01 to 1 µg/mL.

Analyst 1.6.3 software (AB Sciex, Framingham, MA, USA) was used for calculations. Following the Polish Pharmacopoeia VI, the content of an active substance in tablets or capsules should not exceed ±10% (if less than 100 mg of the active substance is declared in one unit) or ±5% otherwise. These requirements apply to medicines. For dietary supplements no exist specific guidelines to assess their quality and take point of the fact that dietary supplements are manufactured in the same form as medicines, the same criteria for the content of Pro were adopted in this study.

### 2.6. Dissolution Test for Tablets or Capsules

The dissolution test was based on Stepien and Giebultowicz [21]. For tablets and capsules, we used USP II Varian VK 7025 and USP I Varian VK 7025 dissolution testers (Erweka GmbH, Heusenstamm, Germany), respectively. We randomly selected six tablets or capsules for each supplement and individually placed them in the dissolution vessels containing 900 mL of dissolution medium. The stirring speed was 50 rpm for tablets and 100 rpm for capsules; the temperature was 37 ± 0.5 °C. Aliquots (1.5 mL) of the medium were manually collected using 5 mL syringes after 30 min of the test and filtered through a Millex-HA 0.45 µm filter. Each aliquot withdrawn was replaced with 1.5 mL of fresh medium. The experiment was performed in 0.1 mol/L hydrochloride acid (simulated gastric conditions) and 0.05 mol/L phosphate buffer (pH 6.8, simulated intestinal conditions).

### 2.7. Expanded Uncertainty

To verify whether the release of Pro is equal to the amount of Pro detected in the dosage unit, we used Equation (1).

(1) $$U\left(x_{1}-x_{2}\right)=2\;\sqrt{{\left[u(x_{1})\right]}^{2}+{\left[u(x_{2})\right]}^{2}}$$

The measurement results were equal if:

$$\left|x_{1}-x_{2}\right|<U\left(x_{1}-x_{2}\right)$$

$x_{1}$—mean [mg] Pro content determined in tablet/capsule (*n* = 3);
$x_{2}\;$—mean [mg] amount of Pro released;
$u\left(x_{2}\right),u\left(x_{1}\right)$—standard uncertainties of the measured values: $x_{1}$ and $x_{2}$ determined according to the formula:

$$u\left(x_{1}\right)=\frac{S}{\sqrt{n}}$$

$S_{}$—standard deviation of the amount of Pro in dosage unit [mg] or standard deviation of the released amount of Pro [mg];
*n*—the number of tablets or capsules used for analysed.

## 3. Results and Discussion

### 3.1. Tentative Contaminants Present in Pro Supplements

In addition to Pro and lysine (an ingredient listed in Pro 7) five contaminants were detected in the analysed supplements. The level of their content was in the range of 0.94% to 7.61% of the Pro area (Table 1). Surprisingly, we have not noticed any transformation products of Prothat can be generated, e.g., during storage.

Among the possible contaminants are substances with various properties. Potentially present in each sample is 4-ethylguaiacol (A1). A1 is a compound naturally occurring in high concentrations in coffee and can be used in industry as a flavour and fragrance [39]. Namely, it is used in vanilla flavour. Its intentional addition to a dietary supplement cannot be excluded. However, it is not included in the composition list as recommended [40]. None of the tested preparations was declared to contain any flavouring agent. Cross-contamination between different production lines is also a likely explanation.

Stearamide (A4) and erucamide (A5) were found in two supplements from the United States. A4 and A5 are fatty acid amides with a wide range of applications, e.g., as anti-adhesive and coating components. In addition, the FDA classifies them as indirect food additives, i.e., substances that are assumed to come into contact with foodstuffs during their packaging, storage, or processing but are not intended to be used as a direct food ingredient [41]. Their presence in dietary supplements may be the result of the manufacturing process or migration from packaging. During the quality analysis, we also detected compounds with similar *m*/*z* and fragmentation as 4-methylpyridine-3-sulfonic acid (A2). A2 is used by industry and is an irritant to mucous membranes and the upper respiratory tract [42]. In the same supplement, a compound with a similar structure to sulfisoxazole was noted (A3). Sulfisoxazole is a pharmacologically active substance belonging to the group of sulfonamides. Compounds of this class are part of the antibacterial drugs currently used to a small extent, mainly topically, because of the resistance of microorganisms and severe side effects, such as allergic reactions and bone marrow damage [43]. Due to its current limited use, cross-contamination during production seems unlikely. Thus, both A2 and A3 can be somehow related to the addition of methylsulfonylmethane (which is labelled an ingredient in Pro 7) to the supplement and be its contaminants and/or products of the reaction between different ingredients.

Here, we suggested only the probable structure of contaminants based on the *m*/*z*, isotopic pattern, and fragmentation. However, identification requires further analysis using comparative standards. Additional to these results, in the literature, there are some reports on the identified impurities, which are considered a significant problem not only in Poland but also in other countries of the world. As an example, tryptophan’s dietary supplements contained some contaminants like tryptophan’s metabolites, condensation products of tryptophan and carbonyl compounds, tryptophan degradation products, degradation products of kynurenine, and other contaminants, e.g., melatonin or glucosamine [21].

One of the contaminants sought and detected by researchers in dietary supplements are mycotoxins [16,18], microbial contaminants [44], pesticides [45], and metals [46,47]. However, they can be found in herbal products, so it makes no sense to determine these contaminants in Pro products. Non-labelled substances may appear in dietary supplements as unintentional impurities and deliberate adulterations with pharmacologically active ingredients. Particularly exposed to their occurrence are preparations from which consumers usually expect immediate and noticeable effects: presented as supporting sexual performance, slimming, or building muscle mass (also physical performance, intended for athletes) [16,48]. It is also not the case with Pro products, which are designed as skin and hair conditioning agents.

Pro contaminants are relatively low (up to 7.6%). Thus, no adverse effects are expected. However, since the quantitative analysis was not performed, no conclusion on the safety of Pro supplements can be drawn.

### 3.2. Determination of Pro in Dietary Supplements

According to manufacturers, analysed supplements contained Pro in the range of 25–520 mg (Table 2). None of the dietary supplements analysed had Pro content within the accepted range for all three dosage forms studied. In the case of Pro3 (CV = 5.9%), Pro6 (CV = 7.2%), and Pro7 (CV = 16.4%) supplements, 1 out of 3 analysed units contained the amount of Pro that met the adopted criteria. The lowest Pro content (75% declared by the manufacturer) was determined for the Pro2 preparation. An insufficient amount of Pro and low variation (CV = 2%) suggest a lower amount of Pro added during product manufacturing rather than an inadequate distribution of the active substance. This may be considered a premeditated act to the consumer’s disadvantage.

In two cases, higher content of Pro than declared was noted. The highest content of Pro (132% of the declared content by the manufacturer) was observed for the Pro7 supplement. The reason may be an incorrect (too high) amount of Pro used during manufacture. The coefficient of variation equal to 16.4% for this supplement also indicates improper mixing of the tablet mass.

To sum up, during the quantitative analysis, we encountered two essential factors: different than declared Pro content and an inhomogeneous mixing of the tablet or capsule mass at the manufacturing stage. The difference between the stated and determined content of the main component has been previously described for tryptophan [21], melatonin [24] and lutein [26] supplements. Twenty out of 22 tryptophan supplements, 10 out of 10 lutein supplements and 7 out of 17 melatonin supplements did not meet the criteria applied in the current work. For dietary supplements with tryptophan, the content of the main ingredient ranged from 55–100% in capsules and 69–87% in tablets. Moreover, low content uniformity was observed [21]. In the case of melatonin or lutein supplements, it is unclear whether the homogeneity of the distribution of the melatonin or lutein among the dosage units was checked. Therefore, it was impossible to determine the quality of these preparations comprehensively.

### 3.3. Dissolution Test for Pro Tablets and Capsules

The Food and Drug Administration (FDA) presents guidelines for testing medicines. Following the requirements concerning the dissolution test for the unmodified release of solid oral medicine, the active substance should be released at 80% or more of the declared content within 30 min of conducting the dissolution test. There is a lack of specific guidelines for conducting release tests in the case of dietary supplements. For this reason, the same criteria for the dissolution test were used for this study. The manufacturers of the analysed dietary supplements did not provide information on the packaging that the preparation has a modified release. Therefore, Pro should be released in the accepted range (above 80%) at pH 1.2 in all analysed dietary supplements. pH 1.2 corresponds to the gastric conditions and in this place of the gastrointestinal tract, Pro should be released from the formulation. Additionally, a dissolution test at pH 6.8 corresponding to the small intestine conditions was performed to verify the potential place of Pro release in the analysed supplements.

**Figure 1:**
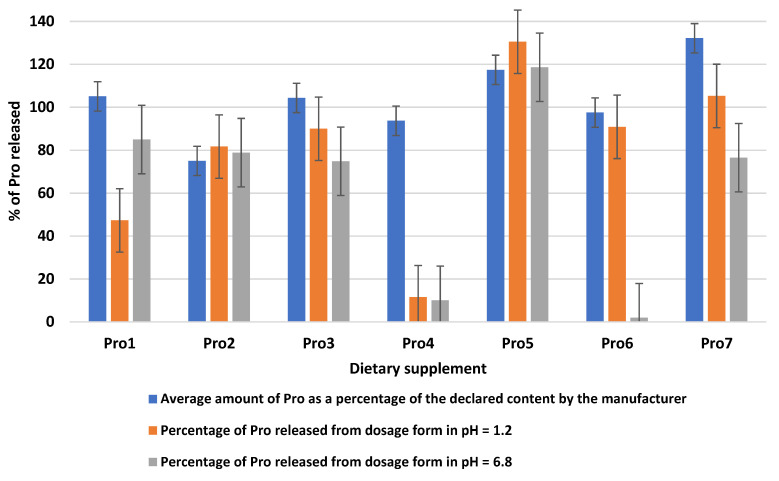
Comparison of the dissolution test results in pH 1.2 (simulated gastric conditions), pH 6.8 (simulated intestinal conditions) with the Pro content analysis for dietary supplements in the dosage form. Comparison of the amount of Pro released and the Pro amount determined in the units, using the extended uncertainty can be found in Table 3.

Among the supplements analysed, we found two that met the criteria at pH 1.2; for Pro3 and Pro5, all units tested (*n* = 6) had Pro release above 80%. For Pro2, Pro6 and Pro7, only the average release was above 80%, with some units releasing less. The release of Pro lower than 50% was noted for two products. However, one of them (i.e., Pro 1) released a high amount of Pro in intestinal pH, indicating that most of the Pro in the product will be available to the consumer. The quality of Pro4 is most questionable. For this formulation, a release of only 11% of the active ingredient in an acidic environment, and 10% in an alkaline environment was reported (Table 3). Given the high Pro content of the supplement (94%), we conclude that the low level of Pro release may have been due to improperly selected manufacturing process parameters and inadequate excipients (Figure 1).

In summary, 2 out of 7 of the Pro supplements released a minimum of 80% Pro (in the case of each analysed capsule or tablet). This means that these preparations met the requirements for releasing the active substance in medicinal products. It is impossible to compare the Pro release data we have obtained with other dietary supplements because supplements containing this amino acid have not been studied. The quality of these supplements is much better than that of tryptophan [21] or lutein [26], as none of these dietary supplements meet these requirements. Low release of active ingredients such as iron, zinc, manganese [49], grape seed extract [30], melatonin [27], folic acid [28,32], and calcium carbonate [31] have also been reported. The reason is the low content of active substances or incorrect technological parameters.

In our study, Pro release was higher from tablets (105%) than from capsules (11–131%). In the literature, we found similar results for tryptophan [21], lutein [26] and folic acid [28]. This indicates that manufacturers have more problems with maintaining quality in the case of capsule formulation.

Qualitative analysis, quantitative analysis and the release of active ingredients from products are essential to ensuring good quality [50]. Dissolution testing measures the amount of active ingredient that passes from the drug into the fluid. It is the basic test for determining the quality of a medicinal product. However, this test is not required for dietary supplements. [51]. This assay can somewhat predict in vivo absorption [25]. A low release rate means low absorption. So even if the active ingredient is present in the dietary supplement in the amount declared by the manufacturer, but the release of this ingredient is low, no physiological effect will be observed.

The lack of a legal framework can be considered the main reason for the low quality of dietary supplements. Since formally they are nutrients (both in Poland and other EU countries [14] and in the United States [52]), they do not have to meet the exact requirements as medicinal products—despite the same form, availability in pharmacies and intensive advertising that may create additional confusion for consumers.

Some European researchers suggest that ensuring the quality of dietary supplements should be based on rules similar to or the same as those for medicinal products, e.g., by introducing the obligation to comply with GMP rules in production, as is the case in the United States [16,53]. This solution combines the guidelines for food with those for medicinal products, including those concerning sanitary conditions, the quality of the operations carried out, the prevention of adulteration, and ensuring the final product’s purity, composition, and quality. Where possible, compliance with the requirements established in official compendia, such as USP [53,54,55], would be expected. It is also considered necessary to raise the awareness of the public, especially healthcare professionals, about the potential benefits and risks of taking dietary supplements [53].

Although there are undeniable dietary supplements on the market that take the consumer into account, the main problem is finding them among preparations of dubious quality, which can also be sold. It is necessary to amend the current legislation, as the ease of marketing and low standard of inspection of dietary supplements make them vulnerable to negligence during production. This poses a threat to the interests and health of consumers. The dissolution test here estimates the amount of proline released from dietary supplements in the gastrointestinal tract. However, the in vitro analysis did not include enzymes and surfactants that can be found in body fluids. Therefore, the in vivo release may be different. Moreover, not all released doses may be absorbed into the circulation and generate a physiological effect. Therefore, the study’s next step should be to determine the bioavailability of proline from supplements. In addition, an important aspect to investigate is the effect of detected impurities using a cytotoxicity assay on cell lines or animals.

## 4. Conclusions

Most of the Pro supplements are of acceptable quality. The number and level of impurities were low. The amount of Pro in one case was 25% lower than declared. In other cases, it was similar or even higher. A more significant problem is the proper preparation of the dosage unit. For example, one supplement may be inactive because a deficient release of Pro was reported. Therefore, a legal framework should be established to promote the appropriate quality of supplements for consumers.

## Figures and Tables

**Table 1:** Tentative* identification of contaminants detected in Pro supplements and their MS parameters.

Code	Formula	Neutral Mass Calculated from the Formula [Da]	Δ Mass [Da]	Δ Mass [ppm]	RDBE ^a^	H/C ^b^	SFit ^c^ [%]	PC ^d^ [%]	X/Pro ^e^ [%]	Fragments [*m*/*z*]	Dietary Supplements Containing Contaminant (% of the Analysed)	Tentative Name
A1	C9 H12 O2	152.08373	0.00002	0.16	4	1.3	81.54	99.48	3.51–7.61	83.04901; 93.06975; 107.08539; 111.04391; 125.05946; 135.07991	All (100%)	4-ethylguaiacol
A2	C6 H7 N O3 S	173.01466	0.00005	0.27	4	1.2	70.72	98.46	2.18	93.05725	Pro7 (14%)	4-methylpyridine-3-sulfonic acid
A3	C11 H13 N3 O3 S	267.06776	−0.00027	−1.01	7	1.2	63.45	100.00	2.53	92.04922; 108.04426; 113.07071; 156.01105	Pro7 (14%)	Sulfisoxazole
A4	C18 H37 N O	283.28751	−0.00002	−0.07	1	2.1	91.29	99.90	5.65	88.07558; 102.09121; 116.10651	Pro3 (14%)	Stearamide
A5	C22 H43 N O	337.33446	0.00003	0.08	2	2.0	65.66	99.82	0.94–1.25	83.08537; 97.10140; 109.10137; 149.13242; 303.30444; 321.31512	Pro3, Pro5 (28%)	Erucamide

^a^ Rings and double bonds equivalent, ^b^ hydrogen versus carbon atoms ratio, ^c^ spectral similarity score between measured and theoretical isotope pattern in %, ^d^ matched intensity percentage of the theoretical pattern, ^e^ % of the main ingredient area, *—identification was performed based on *m*/*z*, isotopic pattern and fragmentation spectra (coverage by in silico fragmentation). The results were not validated using the analytical standard.

**Table 2:** Results of quantitative analysis of Pro in dietary supplements (maximum error value above 20 is bolded).

Code	Dosage Form	Source	Declared Pro Content [mg/unit]	Determined Pro Content [mg/unit] ^a^	Maximum Difference [%]
Pro1	Capsule	Poland	520	546 (CV = 9.9%)	15
Pro2	Capsule	Poland	500	375 (CV = 2.0%)	−27
Pro3	Capsule	United States	500	522 (CV = 5.9%)	9
Pro4	Capsule	Germany	400	375 (CV = 9.8%)	−16
Pro5	Capsule	United States	500	587 (CV = 2.6%)	21
Pro6	Capsule	Poland	270	263 (CV = 7.2%)	−11
Pro7	Tablet	United States	25	33 (CV = 16.4%)	56

CV—coefficient of variation; ^a^—mean (standard deviation *n* = 3).

**Table 3:** Comparison of the results of the amount Pro released from the dosage form and the Pro amount determined in the units using the expanded uncertainty.

Code	The Average Percentage of Pro Amount Released from a Dosage Form (Standard Deviation *n* = 6)			Expanded Uncertainty Parameters				
			pH 1.2			pH 6.8		
	pH 1.2	pH 6.8	$\left|x_{1}-x_{2}\right|$	$U\left(x_{1}-x_{2}\right)$	Equal ^a^	$\left|x_{1}-x_{2}\right|$	$U\left(x_{1}-x_{2}\right)$	Equal ^a^
Pro1	47 (42)	85.0 (7.4)	300.1	187.6	No	104.4	70.1	No
Pro2	82 (13)	79 (16)	33.2	52.3	Yes	19.1	61.9	Yes
Pro3	90.0 (5.2)	74.9 (4.8)	71.8	41.5	No	147.4	40.7	No
Pro4	11 (12)	10 (10)	328.9	57.6	No	334.6	52.9	No
Pro5	131 (30)	118.6 (5.9)	65.4	122.5	Yes	5.8	29.5	Yes
Pro6	91 (12)	2.0 (1.1)	17.8	32.9	Yes	257.9	21.9	No
Pro7	105 (19)	77 (12)	6.7	7.3	Yes	13.9	6.7	No

^a^ Amount of Pro in the formulation and amount of Pro released are equal (yes) or not (no) within the uncertainty.

## Data Availability

Data is contained within the article.
